# Light pollution and habitat fragmentation in the grey mouse lemur

**DOI:** 10.1038/s41598-024-51853-7

**Published:** 2024-01-18

**Authors:** Thomas Le Tallec, Clara Hozer, Martine Perret, Marc Théry

**Affiliations:** UMR 7179 MECADEV, Centre National de la Recherche Scientifique, Muséum National d’Histoire Naturelle, 1 avenue du petit Château, 91800 Brunoy, France

**Keywords:** Behavioural ecology, Conservation biology, Urban ecology, Animal behaviour

## Abstract

Light pollution, by changing organisms’ behavior, affects locomotion, migration and can ultimately fragment the habitat. To investigate the effects of light pollution on habitat fragmentation, we conducted an experimental study on a nocturnal and photosensitive primate, the grey mouse lemur (*Microcebus murinus*). Twelve males were housed individually in an apparatus with two cages connected by two corridors, opaque and transparent. During 4 nights, the transparent corridor was illuminated by specific light intensities: 0 lx, 0.3 lx, 20 lx and 51.5 lx corresponding respectively to total darkness, full moon, minimal intensity recommended by the European standard EN-13201 on public lighting, and to light pollution recorded in an urban area. Each night, general activity, use of corridors and cage occupancy were recorded using an infrared camera. For the first time in a nocturnal primate, results demonstrate that light pollution changes the preference of use of corridors, modifies the locomotor pattern and limits the ability of animals to efficiently exploit their environment according to a light intensity-dependent relationship. However, results indicate that a dark corridor allows partial compensation partly preserving general activities. This study highlights the necessity to consider light pollution during the implementation of conservation plans and the relevance of nocturnal frames.

## Introduction

Habitat fragmentation, which is one major factor implicated in the global decline of populations and species^1–3^, refers to the process of subdividing a continuous habitat into smaller discontinuous patches^4^. Specifically, habitat fragmentation simultaneously involves four processes: (1) reduction in habitat amount; (2) increase in number of habitat patches; (3) decrease in size of patches; (4) increase in isolation of patches^5^. In turn, these processes affect abiotic and biotic habitat conditions and, consequently, modify ecosystem equilibrium. This includes increase in edge habitat in relation to the total area, modification of micro-climate (e.g. changes in wind, temperature or sunlight exposure), affecting both plants and animals distribution (potentially restricting gene flow, leading to an increase in homozygosity and inbreeding because of isolation), but also repercussions on predator–prey relationships and disruption of trophic cascades^6–12^. Depending on species, these changes can have either positive or negative consequences on survival and fitness^13–15^.

Over the past decades, extensive research has investigated habitat fragmentation related mechanisms^16–21^ but only few studies have taken into account the introduction of artificial light in the nocturnal environment and its potential consequences on habitat fragmentation^22–25^. However, light pollution has demonstrable effects on organisms’ behaviour, such as changes in orientation or attraction exerted by the illuminated environment, the ‘flight-to-light’ behaviour of nocturnal insects and migratory birds around artificial light being the best documented effect^26,27^. As a result, these behavioral disturbances can affect locomotion and migration patterns^28^ and contribute to habitat fragmentation. There are two scenarios identified in which light pollution limits locomotion and migration: (1) the ‘captivity’ effect when organisms are disturbed from their normal activity by contact with artificial light and are unable to escape from the proximity of lighting, and (2) the ‘crash barrier’ effect when organisms are disturbed during long-distance movement by artificial light encountered in their travel path. Artificial light prevents organisms from following their original flyway and makes them unable to leave the illuminated environment.

In mammals, few studies have documented habitat fragmentation through the effects of light pollution, whereas it is thought that mammals could be, along with birds, more behaviourally affected by artificial light because of the physical structure of their eye^29^. In a study of dispersing puma (*Puma concolor*), artificial light was detrimental for juveniles exploring new habitat, as they moved away from the urban artificial lights and navigated toward the darkest horizon, especially in a road undercrossing or in open habitats^30^. In bats, light pollution fragmented commuting routes with associated negative conservation consequences. Specifically, slower-flying bats (*Rhinolophus hipposideros* and *Myotis* spp.) avoided artificial light due to light-dependent predation risk^31,32^. Similarly, another study highlighted that nighttime artificial illumination reduced total home size and home range overlap between conspecifics of two mammal species, the bank vole (*Myodes glareolus*) and the striped field mouse (*Apodemus agrarius*)^33^. In a study assessing the effect of artificial light on wildlife use of a passage structure submitted to different light treatments, the authors demonstrated that several mammal species, such as the Columbia black-tailed deer (*Odocoileus hemionus columbianus*), the deer mouse (*Peromyscus maniculatus*) or the opossum (*Didelphis virginiana*) traversed a bridge under-road passage much less when sections or neighbouring sections were lit compared to when none were, suggesting avoidance due to direct or nearby presence of artificial light^34^. These studies point out that light pollution, by changing movement patterns and interindividual interactions, establishes barriers to connectivity on the landscape and can isolate populations. This can result in reducing mammals’ ability to maintain genetic diversity, increasing their susceptibility to disturbance and disease, and limiting their access to resources, potentially leading to fitness consequences on the population level. These studies also highlight the need of finding mitigation solution of artificial light at night, such as under-road unlit corridors, and to examine their efficiency. In primates, the effects of habitat fragmentation on their ecology and biology have been extensively demonstrated^35–37^ but very few studies have explored the impact of light pollution^38,39^. Yet, moonlight has been shown to influence their activity patterns. Lunar philic behaviour seems to be particularly prevalent in nocturnal and cathemeral primates (*Galago*, *Eulemur*, *Tarsius*, *Aotus* species)^40–43^. This behaviour is characterized by an augmentation of travelling and foraging activities in moonlight, attributed to enhanced prey detection. Conversely, other studies have pointed out a lunarphobic behaviour or observed no effect in certain species, including lorises (*Nycticebus pygmaeus*)^44^ and mouse lemurs (*Mirza zaza*)^45^.

Among the nocturnal primates, the grey mouse lemur (*Microcebus murinus*) is a primate belonging to the suborder Strepsirhini and to the Cheirogaleidae family, comprising small, omnivorous primates. Grey mouse lemurs are mostly arboreal and typically forage alone, while they exhibit group sleeping behavior during daylight hours, especially females^46^. Males typically disperse from the natal site before their first mating season, causing a tendency to localized clustering of kin females in these populations^47,48^. They are widely distributed across diverse regions in Madagascar, residing in the dry deciduous forests spanning from southern to northwestern Madagascar, where significant seasonal fluctuations take place^49^. The hot rainy season (“summer”, October to March) is characterized by extended daylight, higher temperatures, and abundant food, leading to increased activity, elevated metabolic rates during the dark phase, and mating behavior. In contrast, the cooler dry season (“winter”, April to September) poses challenges with limited food resources and lower temperatures. As the dry season commences, mouse lemurs adapt by significantly slowing down their metabolism, resulting in increased fat deposits and distinct daily phases of hypometabolism^50,51^. These physiological adjustments are closely tied to the photoperiod^52^. Mouse lemurs inhabit both continuous and fragmented forest patches, with a preference for the interior over edges^53,54^. Given Madagascar's status as one of the most fragmented forest landscapes in the tropics and the documented increase in light pollution in Malagasy protected areas over recent decades^55^, this study aimed at exploring the potential amplification of habitat fragmentation by light pollution in mouse lemurs. We assessed the effect of light pollution on behavioural activities of male mouse lemurs by testing the choice for travelling between a non-illuminated corridor and a corridor illuminated by different light intensities, increasing from full moon to streetlight (from 0.3 lx to 51.5 lx). Due to its strong dependence on photoperiod and the strong inhibitory effect of light on its general activity^56–58^, we predict that male mouse lemurs will prefer the use of the non-illuminated corridor and this preference will increase according to a light intensity-dependent relationship.

## Methods

### Animals

We included 12 adult male grey mouse lemurs (35.5 ± 3.0 months) in this study, all in a long-day photoperiod. In captivity, seasonal variations of behaviours, daily rhythms and physiological functions are entrained by alternating 6-months periods of winter-like short-day photoperiod (SD; light/dark 10:14) and summer-like long-day photoperiod (LD; light/dark 14:10) under artificial light. Mouse lemurs were studied during 4 consecutive nights in mid-LD. They were housed individually at constant ambient temperature (24–26 °C) and relative humidity (55%) with food in excess including a homemade milky mixture (46 kJ/day) and fresh fruits (18.5 kJ/day) delivered every day during the diurnal resting phase and water available ad libitum.

### Ethical note

All experiments were performed in the laboratory breeding-colony of Brunoy (UMR 7179 CNRS/MNHN, France; agreement n° E91-114-1 from the Direction Départementale de la Protection des Populations de l’Essonne) under the authorization n° Ce5/2011/067 from the Charles Darwin Ethics Committee in Animal Experiment and the Internal Review Board of the UMR 7179. All experiments were conducted under personal license to T. Le Tallec (authorization n° A91-621 from the Direction Départementale de la Protection des Populations de l’Essonne) and followed guidelines approved by the Association for the Study of Animal Behaviour and by the Animal Behavior Society^59^. The study is reported in accordance with ARRIVE guidelines (to reduce the number of animals involved in the experiment, animals were used as their own control, outcome measures are clearly defined, statistical analysis is fully detailed and the code is available on the following Figshare repository: [10.6084/m9.figshare.24047478], the species, sex and age of animals are reported, the experimental procedures are described in detail).

### Apparatus

**Figure 1:**
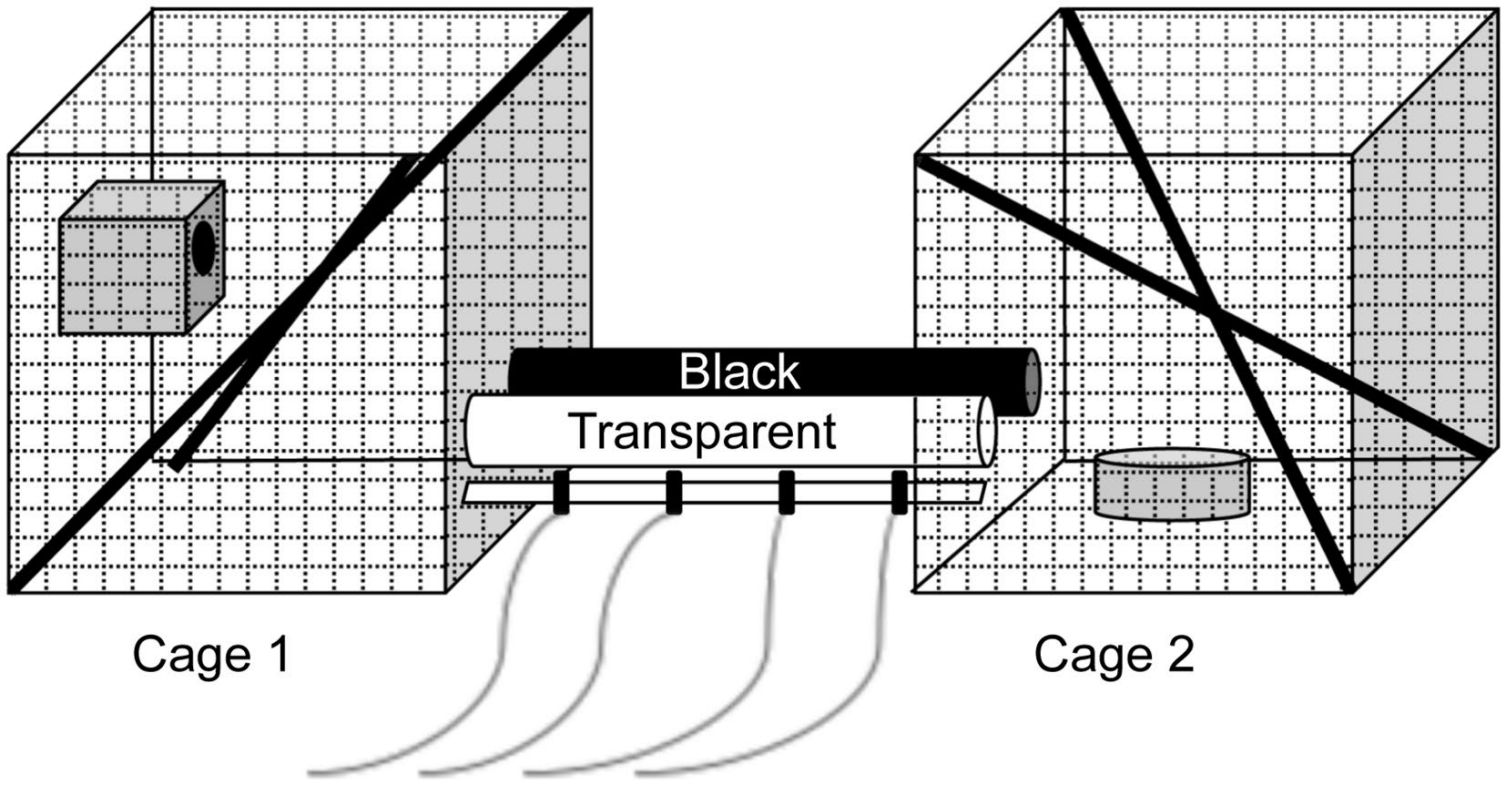
Experimental apparatus. Mouse lemurs were housed individually in an apparatus consisting of two cages (1 and 2) connected by two tubes (black and transparent). The sleeping box was placed in cage 1, whereas food was provided in cage 2. Four yellow LEDs were regularly placed under the transparent tube to illuminate it with different light intensities from 0 to 51.5 lx.

Animals were housed individually in an apparatus consisting of two cages (50 × 50 × 50 cm) connected by two Plexiglas^®^ tubes (respectively opaque and transparent; length: 50 cm; diameter: 8 cm). Cage 1 was enriched with two tree branches and a nest box. Cage 2 was enriched with two tree branches and a feeder. The black opaque tube did not allow the transmission of any light. The transparent tube allowed the transmission of 92% of external light. Consequently, to feed after leaving the nesting box in cage 1, animals needed to cross one of the two tubes to reach cage 2. To rest and leave from cage 2, animals needed to cross again one of the two tubes to return to cage 1 (Fig. 1).

### Experimental protocol

During 4 nights, the transparent tube was illuminated by four yellow LEDs (model L-1503YD, Kingbright, Taipei, Taiwan) regularly distributed every 12.5 cm (Fig. 1). LEDs simulated the irradiance spectra of streetlights with high pressure sodium lamps, the most common artificial light used for outdoor lighting characterized by emission lines from 569 to 616 nm^60^. Night 0 was a habituation period without any light treatment. Each following night, the transparent tube received a different illumination treatment with a specific light intensity: (1) 0 nmol photons s^−1^ m^−2^ in night 1 (control treatment, CTL); (2) 3.5 ± 0.1 nmol photons s^−1^ m^−2^ in night 2 (0.3 lx treatment), corresponding to the intensity of full moon^61^, the most important source of natural light at night^62^; (3) 246.3 ± 0.9 nmol photons.s^-1^.m^-2^ in night 3 (20 lx treatment), corresponding to the minimal light intensity to maintain in urban areas as recommended by the European standard EN-13201–02 on public lighting^63^; (4) 628.0 ± 2.1 nmol photons.s^-1^.m^-2^ in night 4 (51.5 lx treatment), corresponding to the average light intensity of streetlights with high pressure sodium lamps sampled in the city (77 lamps). For each LED, the average light intensity was calibrated before the experiment using a JAZ spectrometer (Ocean Optics, Dunedin, Florida, USA) between 300 and 700 nm. Before the experiment, we randomly determined the initial position of each tube (black or transparent) that was reversed between each light treatment, to prevent any laterality effect. In addition, before the experiment and between each light treatment, both tubes were cleaned to remove any olfactory cues. Daytime light was provided by fluorescent lamps (1 000 lx), placed in the ceiling of the experimental room.

Throughout the experiment, the activity of each animal was filmed all night (10 h) using an infrared camera (Handycam HDR-SR7, Sony, Tokyo, Japan). The camera was fixed to a large tripod which overlooked the two cages and the corridors. We then determined for each light treatment the total number of crossings of both tubes. In addition, we determined for the black tube and the transparent tube separately the respective number of crossings (Nb and Nt) and the speed of crossing (Sb and St in cm s^−1^). The animal was considered to be in a tube when its four paws were seen in it. We also determined for each light treatment the proportion of time spent in each cage. The animal was considered to be in a cage when its four paws were seen in it. Before the experiment, values of body mass were controlled to ensure of their homogeneity (76.8 ± 2.9 g). Daily caloric food intake (CI in kJ) was calculated each day for each animal from the difference between provided and remaining food mass corrected for dehydration.

### Statistical analysis

All analyses were performed with R version 2.14.2 (R Development Core Team, 2001). We performed two mixed linear models (LMMs) using the ‘lmer’ function of the ‘lme4’ package with individuals’ identity as random variable to assess the effects of light treatment (CTL, 0.3 lx, 20 lx or 51.5 lx), tube (transparent or black) and the interaction between light treatment and tube on the number of crossings and on the mean speed of crossing. To select the variables included in our models, we considered a set of four linear mixed models with the following combinations of explanatory factors: an intercept null model with only the random effect; a model with only the ‘Tube’ variable; a model with only the ‘Light treatment’ variable; a model with both ‘Tube’ and “Light treatment” variables; and a model with both ‘Tube’ and “Light treatment” variables as well as their interaction. We ranked these models using the second-order Akaike information criterion with a correction for small-sample-size (AICc), conserving only the model with the lowest AICc, after checking that delta(AICc) > 2 compared to the other models. We further checked the distribution of residuals with ‘qqPlot’ function of the ‘car’ package for LMMs, and with Shapiro tests. The residuals did not comply with normality in the mean speed model, so we used the ‘boxcox’ function of the ‘MASS’ package to estimate an optimal power transformation that maximizes the normality of the transformed variable. Subsequently, we reran the model by applying the power transformation of -1.5 to the mean speed variable. We then ran post-hoc tests using the function ‘emmeans’ of the ‘emmeans’ package with the Tukey correction, in order to determine which specific groups differed from each other. We ran another LMM with individuals’ identity as random variable to assess the effect of light treatment on the proportion of occupancy of cage 2. Finally, we ran a generalized LMM with individuals’ identity as random variable and a poisson error structure to assess the effect of light treatment on the caloric intake. The probability level for statistical significance was *p* < 0.05. All values are presented as means ± standard error of the means.

## Results

### Number of crossings and speed of crossing

**Figure 2:**
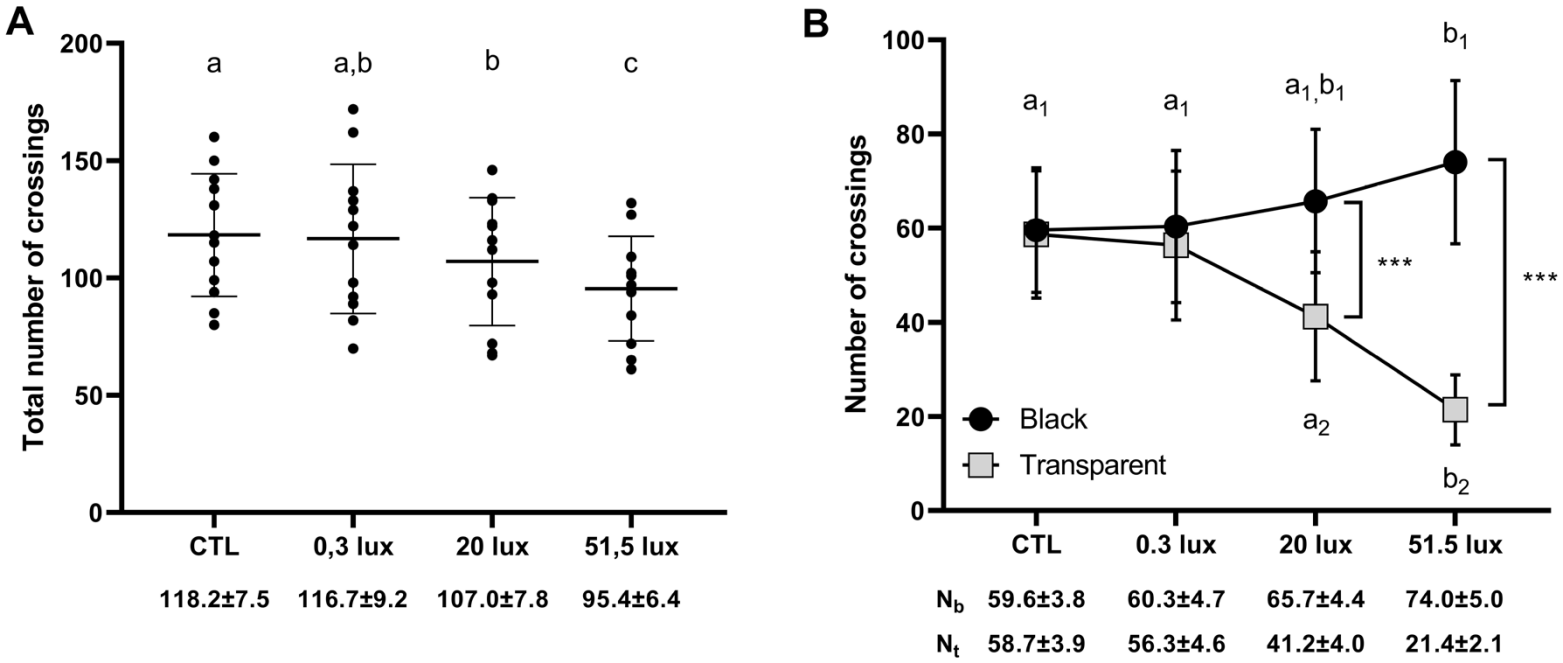
Total number of crossings (**A**) and number of crossings in black and transparent tubes (**B**). The total number of crossings decreased in the 20 lx treatment compared to the CTL treatment and the total number of crossings decreased in the 51.5 lx treatment compared to all the other treatments. Additionally, the number of crossings increased significantly in the black tube and decreased significantly in the transparent tube according to a light intensity-dependent relationship. a-b-c—differences for the total number of crossings between light treatments (CTL, 0.3 lx, 20 lx or 51.5 lx); a_1_,b_1_—significant differences for the number of crossings in the black tube between light treatments; a_2_-b_2_—significant differences for the number of crossings of the transparent tube between light treatments. ***—significant differences (p < 0.001) for the number of crossings between the black tube and the transparent tube. N_b_ = number of crossings in the black tube; N_t_ = number of crossings in the transparent tube.

For both number of crossings and speed of crossing, the statistical models retained included both the ‘Tube’ and “Light treatment” variables as well as their interaction. We observed a significant effect of the light treatment on the total number of tubes crossings (Table 1). The total number of tubes crossings was not significantly different between the CTL and the 0.3 lx treatment, nor between the 0.3 lx and the 20 lx treatments, but was significantly reduced during the 20 lx and 51.5 lx treatments compared to the CTL treatment, and was significantly reduced during the 51.5 lx treatment compared to the 0.3 and 20 lx treatments (Table 2, Fig. 2A). We also observed a significantly higher total number of crossings in the black tube than in the transparent tube, as well as a significant interaction between the tube and the light treatment on the number of crossings (Table 1). While the number of crossings in the black tube was not different between the CTL, 0.3 lx and 20 lx treatments but significantly higher in the 51.5 lx treatment compared to the CTL and 0.3 lx treatments (Table 3, Fig. 2B), the number of crossings in the transparent tube significantly decreased in the 20 lx and 51.5 lx treatments compared to the CTL and the 0.3 lx treatments (Table 3, Fig. 2B). Finally, the number of crossings in the black tube was significantly higher than in the transparent tube in the 20 lx and 51.5 lx treatment but not in the CTL and 0.3 lx treatments (Table 4, Fig. 2B).

**Table 1:** Fixed effects for the model predicting the number of crossings.

	χ^2^	Df	p
Light treatment	40.94	3	** < 0.001**
Tube	207.06	1	** < 0.001**
Light treatment x tube	209.53	3	** < 0.001**

Significant values are in bold.

**Table 2:** Post-hoc test results for differences in the total number of crossings between light treatments.

Contrast	Estimate (SE)	df	t-ratio	p
0.3 lx–20 lx	4.83 (2.01)	77	2.40	0.09
0.3 lx–51.5 lx	10.63 (2.01)	77	5.27	** < 0.001**
0.3 lx–CTL	− 0.79 (2.01)	77	− 0.39	0.98
20 lx–51.5 lx	5.79 (2.01)	77	2.88	**0.03**
20 lx–CTL	− 5.64 (2.01)	77	− 2.79	**0.03**
51.5 lx–CTL	− 11.42 (2.01)	77	− 5.67	** < 0.001**

Significant values are in bold.

**Table 3:** Post-hoc test results for differences in the number of crossings between light treatments for each tube.

Tube	Contrast	Estimate (SE)	df	t-ratio	p
Black	0.3 lx–20 lx	− 5.42 (2.85)	77	− 1.90	0.55
	0.3 lx–51.5 lx	− 13.67 (2.85)	77	− 4.80	** < 0.001**
	0.3 lx–CTL	0.75 (2.85)	77	0.26	1
	20 lx–51.5 lx	− 8.25 (2.85)	77	− 2.90	0.09
	20 lx–CTL	6.17 (2.85)	77	2.16	0.39
	51.5 lx–CTL	14.42 (2.85)	77	5.06	** < 0.001**
Transparent	0.3 lx–20 lx	15.08 (2.85)	77	5.29	** < 0.001**
	0.3 lx–51.5 lx	34.92 (2.85)	77	12.25	** < 0.001**
	0.3 lx–CTL	− 2.33 (2.85)	77	− 0.82	0.99
	20 lx–51.5 lx	19.83 (2.85)	77	6.96	** < 0.001**
	20 lx–CTL	− 17.42 (2.85)	77	− 6.11	** < 0.001**
	51.5 lx–CTL	− 37.25 (2.85)	77	− 13.07	** < 0.001**

Significant values are in bold.

**Table 4:** Post-hoc test results for differences in the number of crossings between black and transparent tubes for each light treatment.

Light treatment	Contrast	Estimate (SE)	df	t-ratio	p
CTL	Black-transparent	0.92 (2.85)	77	0.32	1
0.3 lx	Black-transparent	4.00 (2.85)	77	1.40	0.85
20 lx	Black-transparent	24.50 (2.85)	77	8.60	** < 0.0001**
51.5 lx	Black-transparent	52.58 (2.85)	77	18.46	** < 0.0001**

Significant values are in bold.

**Figure 3:**
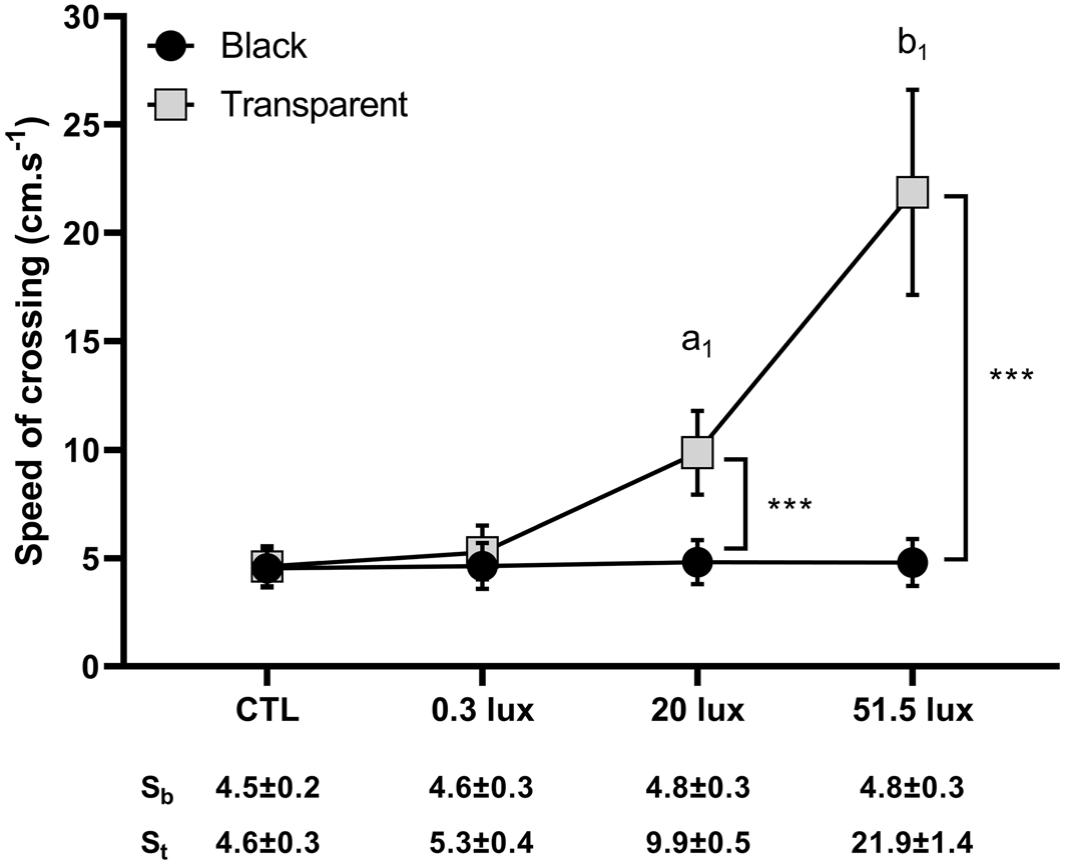
Mean speed of crossing in black and transparent tubes. The mean speed increased significantly in the transparent tube according to a light intensity-dependent relationship but remained constant in the black tube. a_1_,b_1_– significant differences for the mean speed in the transparent tube between light treatments; ***—significant differences (p < 0.001) for the mean speed of crossing between the black tube and the transparent tube. The mean speed of crossing was significantly higher through the transparent tube compared to the black tube in the 20 lx and the 51.5 lx treatments. S_b_ = mean speed in the black tube; S_t_ = mean speed in the transparent tube.

We observed a significant higher mean speed in the transparent tube than in the black tube, as well as a significant interaction between the tube and the light treatment on the mean speed (Table 5). While the mean speed in the black tube was not different between the different treatments (Table 6, Fig. 3), the mean speed in the transparent tube was significantly higher in the 20 lx and 51.5 lx treatments compared to the CTL and the 0.3 lx treatments, as well as in the 51.5 lx treatment compared to the 20 lx treatment, but not between the CTL and the 0.3 lx treatments (Table 6, Fig. 3). Finally, the mean speed in the transparent tube was significantly higher than in the black tube in the 20 lx and 51.5 lx treatment but not in the CTL and 0.3 lx treatments (Table 7, Fig. 3).

**Table 5:** Fixed effects for the model predicting the speed of crossing.

	χ^2^	Df	p
Light treatment	134.58	3	** < 0.001**
Tube	154.12	1	** < 0.001**
Light treatment x Tube	99.50	3	** < 0.001**

Significant values are in bold.

**Table 6:** Post-hoc test results for differences in the speed of crossing between light treatments for each tube.

Tube	Contrast	Estimate (SE)	df	t-ratio	p
Black	0.3 lx–20 lx	− 0.04 (0.06)	77	− 0.64	0.99
	0.3 lx–51.5 lx	− 0.03 (0.06)	77	− 0.54	0.99
	0.3 lx–CTL	0.02 (0.06)	77	0.31	1
	20 lx–51.5 lx	0.01 (0.06)	77	0.10	1
	20 lx–CTL	0.06 (0.06)	77	0.95	0.98
	51.5 lx–CTL	0.05 (0.06)	77	0.85	0.99
Transparent	0.3 lx–20 lx	− 0.63 (0.06)	77	− 10.31	** < 0.001**
	0.3 lx–51.5 lx	− 1.42 (0.06)	77	− 23.15	** < 0.001**
	0.3 lx–CTL	0.13 (0.06)	77	2.07	0.44
	20 lx–51.5 lx	− 0.79 (0.06)	77	− 12.84	** < 0.001**
	20 lx–CTL	0.76 (0.06)	77	12.38	** < 0.001**
	51.5 lx–CTL	1.55 (0.06)	77	25.22	** < 0.001**

Significant values are in bold.

**Table 7:** Post-hoc test results for differences in the speed of crossing between black and transparent tubes for each light treatment.

Light treatment	Contrast	Estimate (SE)	df	t-ratio	p
CTL	Black-transparent	− 0.02 (0.06)	77	− 0.29	1
0.3 lx	Black-transparent	− 0.13 (0.06)	77	− 2.05	0.46
20 lx	Black-transparent	− 0.72 (0.06)	77	− 11.72	** < 0.001**
51.5 lx	Black-transparent	− 1.51 (0.06)	77	− 24.66	** < 0.001**

Significant values are in bold.

### Proportion of cage occupancy

**Figure 4:**
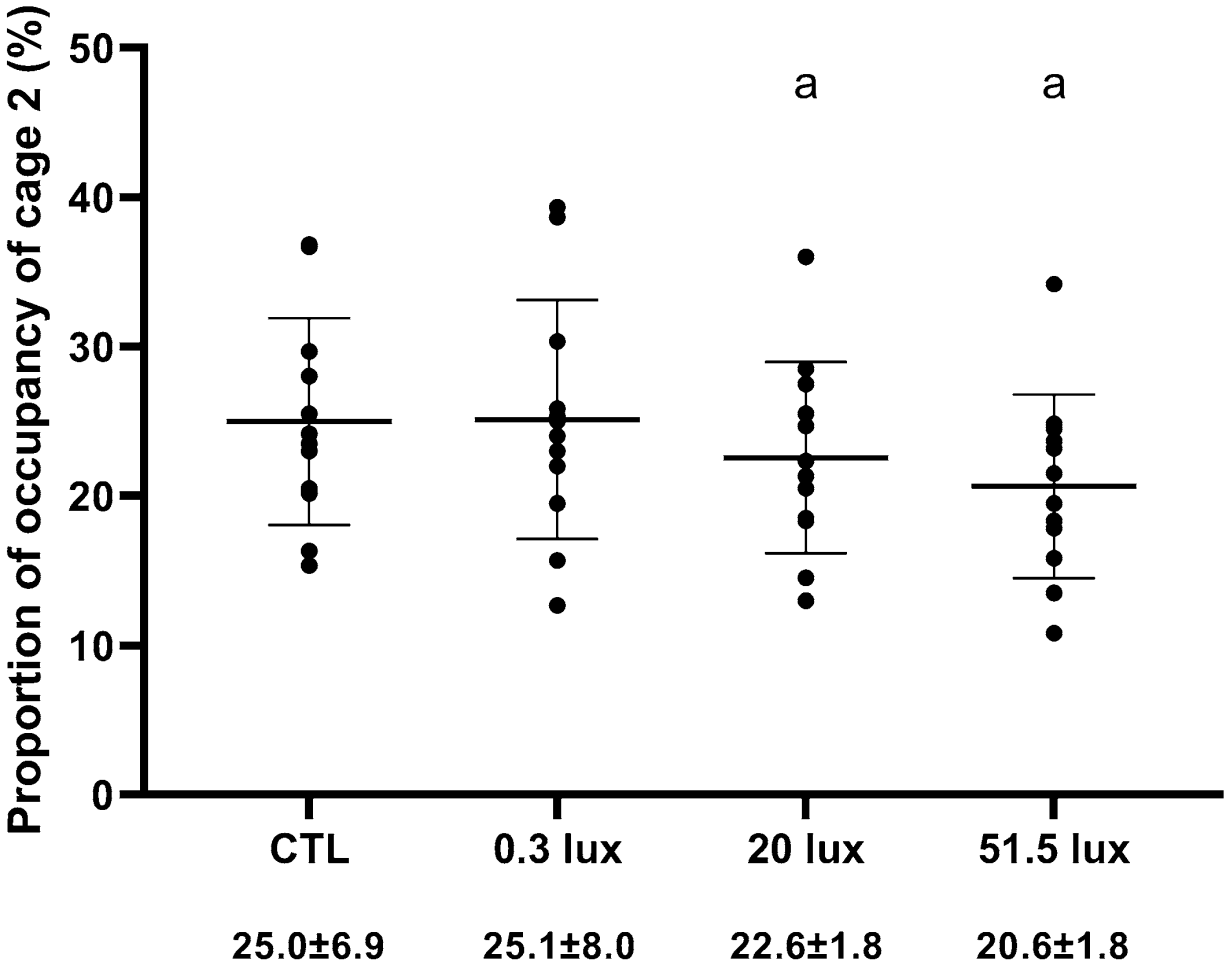
Proportion of time spent in cage 2. Mouse lemurs spent significantly less time in cage 2 during the 20 lx and the 51.5 lx treatments compared to the CTL and the 0.3 lx treatments. a—significant differences for the proportion of time spent in cage 2 between light treatments.

During the nocturnal active phase, male mouse lemurs spent most of their time in cage 1 near their nest box and occasionally moved to cage 2 for feeding. Animals exposed to the CTL treatment spent on average 25% of their time in cage 2 (Fig. 4). We observed a significant effect of the light treatment on the proportion of time spent in cage 2 (LMM: χ^2^ = 39.64, df = 3, p < 0.001). There was no significant difference in time spent in cage 2 between the CTL and 0.3 lx treatments. However, animals spent significantly less time in cage 2 during the 20 lx and 51.5 lx treatments compared to the CTL and 0.3 lx treatments, but we observed no significant difference between 20 lx and 51.5 lx treatments (Table 8, Fig. 4).

**Table 8:** Post-hoc test results for differences in time spent in cage 2 between light treatments.

Contrast	Estimate (SE)	df	t-ratio	p
0.3 lx–20 lx	2.56 (0.83)	33	3.07	**0.02**
0.3 lx–51.5 lx	4.47 (0.83)	33	5.38	** < 0.001**
0.3 lx–CTL	0.14 (0.83)	33	0.17	0.99
20 lx–51.5 lx	1.92 (0.83)	33	2.30	0.12
20 lx–CTL	− 2.42 (0.83)	33	− 2.91	**0.03**
51.5 lx–CTL	− 4.33 (0.83)	33	− 5.21	** < 0.001**

Significant values are in bold.

### Daily caloric food intake

There was no significant difference of daily caloric food intake between the CTL, 0.3 lx, 20 lx and 51.5 lx treatments (GLMM: χ^2^ = 0.32, df = 3, p = 0.95).

## Discussion

In this study, we demonstrate for the first time in a nocturnal primate that light pollution changes the preference of use of corridors, modifies the locomotor pattern, limits the ability of animals to move from one area to another and to efficiently exploit their environment according to light intensity.

Indeed, during the experiment, the total number of crossings through both corridors, illuminated or not, decreased according a light intensity-dependent relationship. Especially, the number of crossings decreased and the speed of crossing significantly increased when using the illuminated corridor. Consequently, light pollution limited the ability to move and changed the locomotor pattern with animals avoiding illumination. In addition, according to light intensity, mouse lemurs reduced the time spent in cage 2, *i.e.* the time allocated to foraging. In wild mouse lemurs, these results suggest that light pollution could affect the connectivity between fragments and limit the ability of animals to move and to effectively exploit their environment and its resources. Similar results have been previously reported in captive male mouse lemurs exposed to light pollution. Indeed, animals under illumination reduced their locomotor activity, spent less time outside their nest box, tended to spend less time feeding outside and brought more fruits into the nest box^39^. However, in both the previous and present study, there was no effect on either the daily caloric food intake or on body mass, most likely because food was provided ad libitum. Nevertheless, in another study, under constant illumination (free-running conditions), animals did not feed at all because of the strong inhibitory effect of light on general activity^56^. Similarly, in the Darwin’s leaf-eared mouse (*Phyllotis darwini*), animals exposed to simulated moonlight carried 40% of their food to the refuge site, consumed 15% less food during the experiment and lost 4.4 g in body mass (around 10% of the mean body weight in this species) in only one trial night^64^. In natural conditions, moonlight has also been shown to negatively affect activity levels of the nocturnal pygmy loris (*Nycticebus pygmaeus*), especially during cold nights^44^. All these responses probably reflected an anti-predator behaviour. In lorises, as it is probably the case in mouse lemurs, the perceived risk of predation increases with illumination of the environment^65,66^. Consequently, to minimize predation risk, preys limit their general activity even at the cost of loss of body mass. In Madagascar, where food availability is highly unpredictable^67^, the impact on body mass could be increased. Besides, exploring the impact of light pollution across different seasons and its interaction with ambient temperature could yield valuable insights. In the dry season, mouse lemurs lower their metabolism, especially on cold nights, and accumulate fat reserves^68^. Consequently, the decrease in activity due to light pollution may be even more significant during this season, because it could potentially increase heat loss, and because the risks associated with predation might outweigh the benefits of hunting, particularly when food is scarce^44^.

Our study suffers from two main limitations. (1) Even though we observed a highly significant impact of light pollution on captive mouse lemurs, it's important to note that this was demonstrated in a single species within a controlled environment. We must exercise caution in extrapolating similar conclusions under natural conditions, where numerous interacting parameters could either diminish or amplify this impact on activity patterns. (2) The experiment was conducted on males but females might respond differently, as observed in wild galagos where moonlight had a positive impact on males' activity levels but not on females^40^. In nature, mouse lemurs exhibit sex-specific activity behaviors, particularly during the cold dry season: for example, while most males remain active with only brief daily periods of hypometabolism, a majority of adult females remain inactive for several months^69^. This suggests that light pollution might have a lesser effect on females compared to males.

### Future implications for conservation policies

In Madagascar, as mouse lemurs become increasingly confined to smaller and isolated forest patches, their population numbers, genetic diversity, and distribution have been altered, sometimes leading to local extinctions^54,70,71^. These detrimental effects of habitat loss and fragmentation could be exacerbated by the increasing levels of light pollution observed in Malagasy protected areas over the past decades^55^. It is therefore crucial to promptly address and mitigate its potential future effects on habitat connectivity. In our experiment, the apparatus provided two corridors. Interestingly, while the number of crossings through the illuminated corridor decreased according to light intensity, it increased proportionally through the black corridor. In addition, the speed of crossing through the black corridor did not change throughout the study. Concretely, when conditions of locomotion through one corridor were not optimal (i.e. illumination), mouse lemurs reversed their preference on the other (i.e. without illumination). However, this compensation was only partial because despite the presence of an optimal corridor, the total number of crossings through both corridors decreased according to light intensity.

This point is crucial for the implementation of conservation plans. For example, in France, the 'green and blue Frame' conservation plan was initiated after a multiparty environmental debate in 2007. The plan aims to establish a network of reserves and corridors nationwide to facilitate communication, movement, feeding, and reproduction of animal species within protected natural areas^72^. However, this plan has been initiated without specific recommendations regarding light pollution. Today, several associations for environmental protection ask for the implementation of a complementary ‘nocturnal frame’ to protect living organisms from the impacts of light pollution (^73^), a provision that has been included in the French Biodiversity Act since 2016 (^74^). Considering that 28% of vertebrates and 64.4% of invertebrates are exclusively or partially active at night, i.e. making them susceptible to disturbances caused by light pollution^75^ and given the widespread nature of light pollution, this inclusion is relevant. In our study light pollution only extended over 50 cm, causing habitat fragmentation on a small scale. However, in a city with 10,000 inhabitants, the sky glow extends up to 20 km and can reach up to 120 km in a city of 1 million inhabitants^76^. Consequently, urban light pollution could lead to habitat fragmentation on a much larger scale. Recently, Mu et al. published an evaluation of light pollution in global protected areas from 1992 to 2018 and reported that there was a significant or a trend of increase in nighttime light in 53% of polluted protected areas in Europe, 78% in South America, and 81% in Africa^77^. In these impacted areas, light pollution, homogenizing the physical environment and being detrimental to photosensitive species, could reinforce biotic homogenization, threaten biodiversity and defeat conservation plans^78^. On the other hand, Japan and the United States of America (USA) exhibit opposite trends (In Japan, 85% of polluted protected areas demonstrated a significant or trend of decrease in nighttime light, while in the USA, the figure stood at 65%), highlighting the significant and positive impact that well-planned ecological conservation policies can have on mitigating light pollution^77^.

Finally, our study shows that light intensities commonly found in urban area or recommended by the European standard EN-13201 on public lighting are sufficient to fragment the habitat in mouse lemurs. This point demonstrates the necessity to find a trade-off between human needs and environmental protection, by implementing public lighting policies, urbanization plans and conservation plans that take the light pollution factor into account. This study also requires broadening the investigation to include a wider variety of nocturnal species, allowing us to generalize our conclusions on a larger scale.

## Data Availability

The data generated and analysed during the current study are available in the Figshare repository [10.6084/m9.figshare.24047475].
